# Pathological Consequences of Drug Abuse: Implication of Redox Imbalance

**DOI:** 10.1155/2019/4780852

**Published:** 2019-09-22

**Authors:** Stefania Schiavone, Margherita Neri, Brian H. Harvey

**Affiliations:** ^1^Department of Clinical and Experimental Medicine, University of Foggia, Foggia, Italy; ^2^Department of Morphology, Surgery and Experimental Medicine, University of Ferrara, Ferrara, Italy; ^3^Department of Pharmacology, Center of Excellence for Pharmaceutical Sciences, North West University, Potchefstroom, South Africa

The onset, progression, and outcome of numerous pathological conditions, affecting different organs and systems, have been widely reported to be significantly impacted by the abuse of psychoactive compounds. In the last decades, preclinical and clinical reports have contributed to a lively scientific debate on the possible pathogenic role that redox imbalance, defined as a disequilibrium between reactive oxygen species (ROS) generating and degrading systems, plays in this scenario [1]. Moreover, increasing interest has focused on the possibility that enhanced ROS production or decreased antioxidant defenses in different body compartments, such as the blood, central nervous system (CNS), cardiovascular, gastrointestinal, and respiratory apparatuses may represent reliable biomarkers that will enable the detection of both the early phases of drug abuse-associated complications and the response to pharmacological treatments. Indeed, in a recent review where the authors attempted to assemble a biomarker panel for mood and psychotic disorders, it was clear that disordered redox forms an integral component of the mood-psychosis continuum [2]. Since prolonged substance abuse invariably leads to the development of a mood and/or psychotic disorder, it is understandable that targeting redox pathways may offer beneficial alternatives to traditional treatment interventions in such conditions [3].

In this special issue, a team of international experts presents their preclinical and clinical findings related to the impact of redox imbalance on pathological conditions associated with the abuse of psychoactive compounds, describing different underlying mechanisms and also highlighting the possibility of translating their results into the development of more targeted pharmacological interventions.

The pathological consequences of ethanol (EtOH) consumption have been widely reported, also that it is considered one of the oldest and most intoxicating psychoactive compounds still being used and abused by humans. Among the different mechanisms proposed to explain the toxicity of this substance, its potential to induce the production of ROS in several body tissues and compartments has been confirmed and extended by several lines of preclinical and clinical evidence [4].

In this context, D. Pamplona-Santos and coauthors performed a study on the effects of serious and episodic EtOH drinking patterns, comparable to weekend utilization. The acute consumption of EtOH promotes an imbalance in CNS metabolic functions, resulting in neurodegeneration and cerebral dysfunctions. In this study, the authors investigated the effects of physical training on a treadmill versus the deleterious effects of EtOH on hippocampal functions related to memory and learning. They demonstrate that physical exercise contributes to the reestablishment of the redox status by elevating GSH levels in the blood and hippocampus, and that exercise is a significant nonpharmacological intervention for the prevention of cognitive dysfunctions caused by EtOH exposure following a binge drinking pattern of consumption.

Importantly, epidemiologic studies have highlighted enhanced EtOH consumption among specific subpopulations, such as women [5] and/or adolescents [6]. With respect to this issue, by using a preclinical approach for their research, L. M. P. Fernandes et al. investigated the impact of moderate EtOH consumption in female rats on oxidative damage-related biomarkers in the liver, brain (motor cortex), and blood, as well as on behavior. These authors reported that repeated EtOH binge drinking in female rats during adolescence was able to induce lipid peroxidation in the brain and liver, where steatosis and structural disruption of the parenchyma were also detected, although no evidence of systemic oxidative damage was found. Moreover, EtOH-induced damage in the brain and liver was accompanied by significant locomotor dysfunction, viz. motor incoordination, even following a single episode of binge-like EtOH exposure. However, bradykinesia and decreased spontaneous exploration required more prolonged EtOH consumption. The authors conclude that their paper questions the adequacy of lipid peroxidation as a reliable biomarker of the detrimental effects of EtOH abuse, at least in the female gender and during the adolescent period, considering the significant vulnerability of the brain and liver to oxidative damage, even in the absence of a systemic ROS increase.

Psychedelic substances have been the object of increasing interest with respect to the possible mental and physical pathological consequences related to their consumption. One of the most widely abused and addictive compound belonging to this class is methamphetamine (METH), which has been reported to induce episodic and/or permanent neuropsychiatric conditions. Moreover, prolonged abuse of this substance is associated with neurotoxicity as well as damages to other peripheral organs. Nevertheless, despite the significant efforts of the scientific community to better understand the complexity of the molecular mechanisms underpinning METH toxicity, several aspects of this process remain to be elucidated. In this context, the review by F. Limanaqi and coauthors discussed the epigenetic effects caused by METH. The manuscript reports the most important molecular events, starting at the presynaptic dopamine terminals to reach the nucleus of postsynaptic neurons. They describe how specific neurotransmitters and signaling cascades produce persistent genetic modifications that enable the shift of neuronal phenotypes to induce alterations in behavior. In the postsynaptic neurons, epigenetic effects induced persistent changes, including sensitization and desensitization, priming, and shift of neuronal phenotype.

As noted earlier, METH induces the production of a number of ROS that leads to lipid peroxidation, protein misfolding, and nuclear damage in the CNS that are detrimental to axon terminals and cell bodies. The overproduction of oxidized proteins, lipids, and nucleic acids requires cellular clearing systems for detoxification and elimination. Cell clearing pathways such as ubiquitin proteasome (UP) and autophagy (ATG) are two such powerful defense mechanisms [7]. However, their integrity and function are challenged by METH administration. Fortunately, the cell clearing organelle, “autophagoproteasome” (APP), possesses both ATG and UP components. Moreover, this organelle is purported to be activated by the mammalian target of rapamycin (mTOR), thus offering a potential pharmacological target for circumventing the actions of METH toxicity. In their paper, G. Lazzeri et al. dissect the ultrastructural morphometry of both UP and ATG components in different cell compartments and, apart from strengthening the concept that mTOR inhibition and ATG protect against METH toxicity, they provide further detail as to the significance of specific ATG-related structures. While the paper contributes towards a better understanding of the neuromolecular processes governing METH toxicity, their findings also speak towards novel insight into cell clearing pathways to counteract several kinds of oxidative damages, as well as hint at new pharmacological strategies for treating METH-associated toxicity.

The pathological impact of ketamine, a N-methyl-D-aspartate (NMDA) receptor antagonist, on mental and physical health has been largely demonstrated by preclinical and clinical evidence. Ketamine is widely recognized in the treatment of resistant depression, although its penchant to induce psychedelic side effects and possible addiction limits its general use. This has prompted the search for alternative treatments or approaches that may abrogate ketamine's psychedelic effects. Among the different molecular mechanisms proposed to explain the detrimental effects of this psychedelic compound, oxidative stress has been reported to play a crucial role [8], which hints at the possible use of antioxidants as an adjunctive treatment when using ketamine. With respect to this topic, S. de Carvalho Cartágenes et al. investigated the effects on oxidative status and behavior after immediate withdrawal of intermittent ketamine administration in adolescent female rats. Although studies exploring gender differences in ketamine responses are limited, they demonstrate that females are much more sensitive than males to the effects of this drug [9]. The data reported by the authors showed that immediate ketamine withdrawal in the adolescence period promotes systemic and hippocampal oxidative stress, and this was accompanied by alterations in emotional behavior.

In recent years, opioid use has approached epidemic proportion, especially in some countries of Europe and North America. An increasing number of evidence has reported a pathological link between opioid addiction and redox dysregulation in both the CNS and periphery [10]. With respect to opioid compounds generally used as substitutes in the maintenance treatment for heroin addiction, such as buprenorphine and methadone, limited lines of evidence are available concerning their possible impact on the redox status. In this context, the clinical approach presented by C. Leventelis and coworkers described increased levels of redox biomarkers and reduced antioxidant defense in blood samples obtained from buprenorphine-treated patients compared to healthy subjects. The same was also observed in subjects receiving methadone, whose impact was even more significant than that of buprenorphine. These findings are important as they suggest that opioids, such as buprenorphine and methadone, that are used to treat opioid addiction, also impact a redox regulatory process in a similar manner as do the more addictive opioid drugs for which they are being used as an intervention strategy against addiction. The authors conclude that their work highlights the possibility of a concomitant administration of antioxidant compounds with the maintenance therapy for heroin addiction. From the presented findings, reflection is needed in the attempt to further elucidate the link between opioid addiction and redox dysregulation, especially regarding the effects of heroin itself and the possibility that buprenorphine and methadone also independently impact on the cellular redox systems. The latter actions displayed by buprenorphine and methadone could underplay their own addictive potential.

In conclusion, this special issue has confirmed and extended the pathological role of disordered redox systems in a variety of pathological conditions, including EtOH, METH, and opioid abuse, as well as the psychedelic substance, ketamine. These findings have been provocative to understanding how apparently different types of neuro- and psychopathology induced by a broad array of psychotropic substances ultimately impact cellular redox systems. Identifying the source of redox disturbance, e.g., ubiquitin proteasome (UP) and autophagy (ATG), as well as a putative pharmacological target, e.g., mTOR, may provide answers how to best treat a certain disorder presenting as a pro-oxidative state. While oxidative stress being prevalent in so many distinct illnesses questions the usefulness of redox parameters as a disease-specific biomarker [2], it does not lessen the importance of targeting these systems to enable a better therapeutic outcome through the use of adjunctive antioxidants in treating conditions varying from mood and psychotic disorders to addiction. However, to better enable this approach requires a thorough understanding of the redox processes involved, in which this special issue has sought to reveal.
